# Job vacancies at the European Centre for Disease Prevention and Control (ECDC)

**DOI:** 10.2807/1560-7917.ES.2020.25.29.2007232

**Published:** 2020-07-23

**Authors:** Eurosurveillance editorial team

**Affiliations:** 1 European Centre for Disease Prevention and Control (ECDC), Stockholm, Sweden

The European Centre for Disease Prevention and Control (ECDC) invites applications for the following positions:

 

**Expert Microbiology**
**Expert Biostatistics**
**Scientific Officer Bioinformatics**

The application deadline expires on 28 Aug 2020.

**Scientific Officer Mathematical Modelling**

The application deadline expires on 21 Aug 2020. 

 

For more information, please visit the ECDC website: 

https://ecdc.europa.eu/en/work-us/vacancies?f%5B0%5D=deadline_date%3A1

